# Immunoprotective Activity Induced by Leptospiral Outer Membrane Proteins in Hamster Model of Acute Leptospirosis

**DOI:** 10.3389/fimmu.2020.568694

**Published:** 2020-10-30

**Authors:** Aline F. Teixeira, Maria F. Cavenague, Leandro T. Kochi, Luis G. Fernandes, Gisele O. Souza, Antonio Francisco de Souza Filho, Silvio A. Vasconcellos, Marcos Bryan Heinemann, Ana L. T. O. Nascimento

**Affiliations:** ^1^ Laboratorio de Desenvolvimento de Vacinas, Instituto Butantan, Sao Paulo, Brazil; ^2^ Programa de Pos-Graduacao Interunidades em Biotecnologia, Instituto de Ciencias Biomedicas, Universidade de Sao Paulo (USP), Sao Paulo, Brazil; ^3^ Laboratorio de Zoonoses Bacterianas, Departamento de Medicina Veterinaria Preventiva e Saude Animal (VPS), Faculdade de Medicina Veterinaria e Zootecnia, Universidade de Sao Paulo, Sao Paulo, Brazil

**Keywords:** *Leptospira*, leptospirosis, vaccine, recombinant proteins, immunoprotection, immunogenicity

## Abstract

Leptospirosis is a zoonotic disease of worldwide distribution, affecting both humans and animals. The development of an effective vaccine against leptospirosis has long been pursued but without success. Humans are contaminated after direct contact with the urine of infected animals or indirectly by contaminated water or soil. The vaccines available consist of inactivated whole-bacterial cells, and the active immunoprotective antigen is the lipopolysaccharide moiety, which is also the basis for serovar classification. However, these vaccines are short-lasting, and protection is only against serovars contained in the preparation. The search for prevalent antigens, present in pathogenic species of *Leptospira*, represents the most cost-effective strategy for prevention of leptospirosis. Thus, the identification of these antigens is a priority. In this study, we examined the immunoprotective effect of eight leptospiral recombinant proteins using hamster as the challenge model. Animals received subcutaneously two doses of vaccine containing 50 *μ*g of each recombinant protein adsorbed on alum adjuvant. Two weeks after the booster, animals were challenged with virulent leptospires and monitored for 21 days. All proteins were able to induce a specific immune response, although significant protective effects on survival rate were observed only for the proteins Lsa14, rLIC13259, and rLIC11711. Of these, only rLIC13259 and rLIC11711 were found to be highly prospective in promoting renal clearance. The sterilizing potential of both proteins will be further investigated to elucidate the immunoprotective mechanisms involved in leptospirosis control. These are the first proteins involved with human complement components with the capacity to protect against virulent challenge and to eliminate the bacteria from the host.

## Introduction

Pathogenic *Leptospira* are the etiological agent of leptospirosis. The disease is globally distributed, and it affects humans and animals. In developing tropical countries, leptospirosis is associated with poor sanitation conditions, while in developed countries, the disease is related to sport and recreational activities (1–3). Rats, especially the brown rats (*Rattus rattus* or *Rattus norvergicus*), are the main reservoir of leptospires. These animals are asymptomatic carriers, carrying and shedding the bacteria through their urine, contaminating water and soil (4–6). Humans and animals are contaminated directly through the urine of rats or indirectly *via* contaminated environments. Humans are accidental and terminal hosts of the disease. The symptoms of leptospirosis are non-specific, ranging from mild flu-like to severe kidney, liver, and lung diseases (7). This diversity of symptoms found in individuals infected with *Leptospira* promotes an increase in the number of misdiagnosed cases, leading to underestimated case numbers (7, 8). A systematic review reported that leptospirosis is responsible for more than 1 million cases per year and approximately 60 thousand deaths (5). The prevention of leptospirosis is crucial for both preventing an increase in disease rate and disrupting the transmission cycle.

Prophylactic measures, such as vaccines, are the best approach to fight infectious diseases. Veterinary vaccines against leptospirosis are available; these are killed or inactivated whole-cell vaccines, which rely on lipopolysaccharide (LPS) content. These vaccines are short-lasting, require re-vaccination after 1 year, and protect only against serovars contained in the preparation. To date, more than 300 serovars are described for pathogenic *Leptospira*. Most existing vaccines contain 3 to 10 predominant serovars of the region, lacking the coverage for potential endemic serovars (8, 9). A cost-effective and long-term protective vaccine against leptospirosis has been pursued by several investigators around the world. It is anticipated that conserved antigen-based vaccines are an attractive alternative to overcome the limitations of current vaccines.

Surface-exposed molecules of pathogens are considered interesting targets for vaccine development due to their cellular location and possible role in host-pathogen interactions. In the last years, several recombinant vaccines containing outer membrane proteins, conserved among pathogenic leptospire species, which act as potential virulent factors, have been evaluated in animal challenge models (10–14). These vaccines have demonstrated a diverse range of protection, dependent on the adjuvant, administration route, and animal model used (15). Leptospiral immunoglobulin-like proteins, known as LigA and LigB, remain the most studied vaccine candidates among leptopiral antigens. However, conflicting results have been reported, and some have demonstrated that these vaccines do not prevent renal colonization (10, 15–18). Thus, the search for new vaccine antigens that are serovar-independent and have the ability to inhibit leptospire dissemination is still necessary. Certainly, the identification of these antigens will contribute to the understanding of the immune mechanism involved in protection against leptospirosis.

In the present study, we evaluated the vaccine potential of eight recombinant proteins, previously characterized as conserved immunogenic proteins, localized at the cell surface and able to interact with several host components. The Lsa25.6 and Lsa16 proteins interacted with laminin, plasminogen, generated plasmin, and fibrinogen, but only Lsa25.6 inhibited fibrin clotting (19). Lsa16 was also able to interact with the mammalian cell receptor E-cadherin. Lsa19, Lsa14, and Lsa24.9 showed concentration-dependent binding with laminin and plasminogen. Moreover, they were able to generate plasmin in the presence of a plasminogen activator, and immunogenic epitopes are believed to be involved in the interaction (20, 21). Interestingly, LipL46, an overexpressed protein of virulent *Leptospira*, showed the capacity to interact only with plasminogen (22). In contrast to LipL46, the recombinant protein rLIC11711 showed binding to a broad range of molecules. It was able to interact with laminin, E-cadherin and collagen IV, and bound plasminogen from normal human serum, interacting with fibrinogen, fibronectin, and components of the complement system (23). The rLIC13259 protein was also characterized as a wide-range adhesin, interacting with laminin, plasminogen, and terminal complement components, mediating the binding of purified or normal serum-derived vitronectin, C7, C8, and C9 (24). These features suggests that Lsa25.6, Lsa16, LipL46, Lsa14, Lsa19, Lsa24.9, rLIC13259, and rLIC11711 leptospiral recombinant proteins may have multifunctional roles in *Leptospira* and thus prompted us to investigate their role to induce a protective immune response in a hamster model of leptospirosis.

## Material and Methods

### Bacterial Strains 

The virulent* Leptospira interrogans* serovar Kennewicki strain Pomona Fromm (LPF) was cultured at 28°C under aerobic conditions in liquid Ellinghausen-McCullough-Johnson-Harris (EMJH) medium (Difco, BD, Franklin Lakes, NJ, USA) containing 10% (vol/vol) rabbit serum. Virulent leptospiral cultures are routinely maintained by infection of golden Syrian hamsters and subsequent bacterial isolation from kidney. *Escherichia coli* BL21 DE3 Star pLysS, BL21 DE3, and BL21-SI cells were used as recombinant protein expression hosts.

### Recombinant Proteins Expression and Purification

Expression and purification of Lsa25.6, Lsa16, LipL46, Lsa14, Lsa19, Lsa24.9, rLIC11711, and rLIC13259 proteins are detailed in the following reports: Pereira et al. (19), Santos et al. 2018 (22), Figueredo et al. (20), Rossini et al. (21), Kochi et al. (23), and Cavenague et al. (24). Briefly, expression of recombinant proteins Lsa25.6 and Lsa16 was performed in *E. coli* BL21 (DE3) Star pLysS expression strains with 0.1 mM isopropyl-β-1-D thiogalactopyranoside (IPTG) for 3 h. Recombinant proteins were purified from the insoluble fraction and they were refolded on-column by gradually removing urea until reaching a concentration of 0 M (19). The LipL46 recombinant protein was expressed in *E. coli* BL21-SI cells with 500 mM NaCl for 3 h. LipL46 was purified from the soluble fraction of *E. coli* lysates (22). Expression of the recombinant proteins Lsa14 and Lsa19 was performed in *E. coli* BL21 (DE3) Star pLysS with 0.1 mM IPTG and *E. coli* BL21-SI with 500 mM NaCl, respectively. Lsa14 was purified from the insoluble fraction, while Lsa19 was purified from the soluble fraction. Lsa14 was refolded in a column by gradually removing urea (20). Lsa24.9 was expressed as inclusion bodies in *E. coli* BL21 (DE3) Star pLysS with 1mM IPTG for 3 h. Urea removal was performed by dilution prior to protein purification (21). After adding 1 mM IPTG, rLIC11711 and rLIC13259 proteins were expressed in the soluble fraction in *E. coli* BL21 (DE3) Star pLysS and *E. coli* BL21 (DE3) strains, respectively. Purification was performed using *E. coli* lysate supernatants (23, 24). All proteins were purified using a Ni+2-chelating chromatography column, and protein concentration was determined using bovine serum albumin as the standard.

### Animal Immunization and Challenge Assays

Male hamsters (6–8 weeks old) were immunized subcutaneously with a 500-*μ*l dose containing 50 *μ*g of each recombinant protein mixed with 12.5% Alhydrogel [2% Al(OH)3] as adjuvant. One booster injection was given after 2 weeks with the same preparation of recombinant protein. In the negative control group referred to as control, hamsters were injected with phosphate-buffered saline (PBS) plus 12.5% Alhydrogel. A group consisting of animals immunized with a dose of 10^9^ heat-killed whole-leptospire referred to as bacterin was included as positive control for survival. Bacterin used in this experiment was prepared in-house as described in Silva et al. (17). Briefly, *Leptospira interrogans* serovar Kennewicki strain Pomona Fromm were harvested by centrifugation and washed pellets were heat-inactivated 56°C for 20 min. Inactivated cells were resuspended in PBS, aliquoted, and stored at −20°C. Two weeks after last immunization, animals were challenged intraperitoneally with an inoculum of 10^4^ virulent leptospires (25). Hamsters received water and food* ad libitum* and were monitored daily for 21 days for clinical signs of leptospirosis. Hamsters surviving after this time were euthanized following guidelines for the euthanasia of animals. Firstly, administration of acepromazine (5 mg/kg) was used as sedative. After 20 min, hamsters received an injection intraperitoneal of ketamine (200 mg/kg) and xylazine (10 mg/kg) for full anesthesia effect and euthanasia was performed by isoflurane inhalation into specific chamber. Both animal kidneys were removed aseptically, macerated, and inoculated in liquid EMJH medium in a dilution 10^−2.^ Bacteria growth was monitored by using dark-field microscopy for up to 1 month. After this time, cultures were designated as positive or negative for the presence or absence of leptospires, respectively. Animals that did not survive the challenge were not included in calculation because their kidneys were not removed to perform culture, but it is expected that they have died out of leptospirosis. Hamsters in each group were bled from the retro-orbital plexus after administration 10 *μ*l of topical anesthetic proxymetacaine hydrochloride 5mg/ml (Anestalcon). Blood collection was performed 1 day before each immunization/challenge and collected serum was kept at −20°C until use. Two independent experiments were performed, each one containing six animals per group. Fisher's exact test was used to compare survival curves between experimental groups, and p<0.05 was considered statistically significant.

### Evaluation of the Humoral Immune Response

Sera of immunized animals were pooled from three animals and analyzed in triplicate by ELISA to determine the production of total IgG antibody and its subclasses IgG1 and IgG2/IgG3. ELISA plates were coated with 250 ng of each recombinant protein, and wells were blocked with PBS-T containing 10% non-fat dry milk and incubated with different dilutions of hamster sera, ranging from 1:200 to 1:12,800. Plates were washed and incubated with either HRP-conjugated anti-hamster total IgG (1:5,000, Sigma), anti-hamster IgG1, or anti-hamster IgG2/IgG3 (1:5,000, Southern Biotechnology). The wells were washed three times, and a solution 1 mg/ml o-phenylenediamine in citrate phosphate buffer (pH 5.0) plus 1 *μ*l/ml H_2_O_2_ was added (100 *μ*l per well). The reaction was allowed to continue for 10 min and was interrupted by the addition of 50 *μ*l of 2 M H_2_SO_4_ to the reaction mixture. Readings were taken at 492 nm with a microplate reader (Multiskan EX; Thermo Fisher Scientific, Helsinki, Finland). The graphs were plotted with average absorbance values found at 200 times serum dilution.

### Ethics Statement

The Ethics Committee for Animal Research of Instituto Butantan approved the use of the animals involved in these studies under protocol number 3549261016. The Committee on Animal Research adopts the guidelines of the Brazilian College of Animal Experimentation.

## Results

### Representative Scheme of Predicted Coding Sequences, Expression, and Purification of Recombinant Proteins

**Figure 1:**
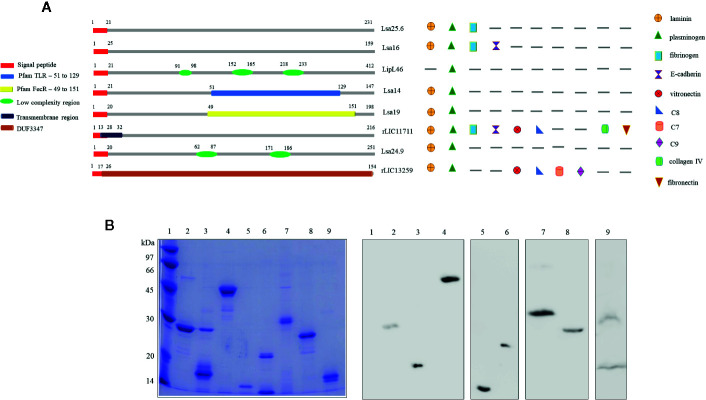
Scheme of predicted coding sequences and purification analysis of each recombinant protein. **(A)** Left panel: the diagram represents the linear sequence of the proteins and its respective conserved domains and/or features, predicted by amino acids sequence analysis. The presence signal peptide sequence shown in red is representative, all genes were cloned without this portion. Right panel: the diagram represents the extracellular matrix and plasma molecules that bound to the respective recombinant proteins, experimentally characterized *in vitro*. **(B)** Sodium dodecyl sulfate polyacrylamide gel electrophoresis (SDS-PAGE) analysis of purified recombinant proteins and immunoblotting probed with anti-his tag antibody. Lane 1: molecular mass protein marker; lane 2: Lsa25.6; lane 3: Lsa16; lane 4: LipL46; lane 5: Lsa14; lane 6: Lsa19; lane 7: Lsa24.9; lane 8: rLIC11711 and lane 9: rLIC13259.

Predicted coding sequences (CDS) LIC13059, LIC10879, LIC11885, LIC11122, LIC12287, LIC11711, LIC10920, and LIC13259 were identified by analyzing the genome sequences of *L. interrogans* serovar Copenhageni (26). The genes LIC13059 (Lsa25.6), LIC10879 (Lsa16), LIC11885 (LipL46), LIC11122 (Lsa19), LIC12287 (Lsa14), LIC11711 (rLIC11711), LIC10920 (Lsa24.9), and LIC13259 (rLIC13259) were cloned as described in Pereira et al. (19), Santos et al. (22), Figueredo et al. (20), Rossini et al. (21), Kochi et al. (23), and Cavenague et al. (24) without signal peptide sequence. A representative scheme of the protein sequences containing their conserved domains and the ligands that were found to interact with the recombinant proteins are shown in **Figure 1A**. Plasmids containing each gene were used to transform *E. coli* expression strains and recombinant proteins were expressed with a 6-his tag and purified by immobilized-metal affinity chromatography as described before. An aliquot of each purified protein was analyzed by sodium dodecyl sulfate polyacrylamide gel electrophoresis (SDS-PAGE) and immunoblotting by using his-tag monoclonal antibody (**Figure 1B**). All proteins showed expected sizes of 25.6, 16, 46, 14, 19, 24.9, 22.8, and 17 kDa, respectively. Lsa24.9 has shown an estimated molecular mass of 24.9 kDa, but on SDS-PAGE it has migrated near the 30 kDa protein standard. Each recombinant protein was dialyzed against PBS prior to injection into animals.

### Evaluation of Humoral Immune Response Induced in Animals Immunized With Recombinant Proteins

**Figure 2:**
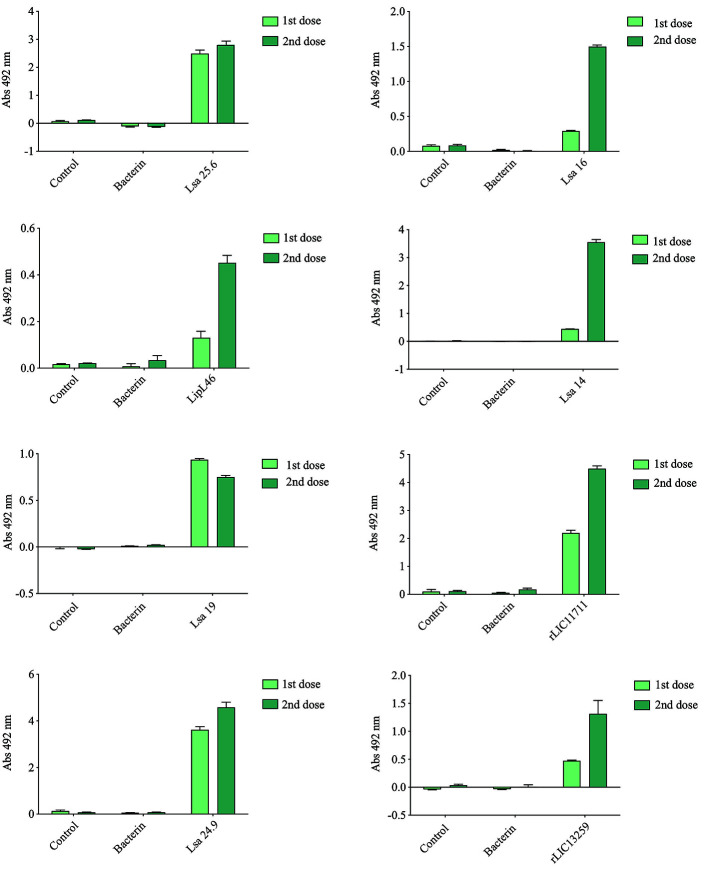
Total IgG production after animal immunization. Hamsters were immunized subcutaneously with emulsions of recombinant protein plus Alhydrogel, twice at 2 weeks intervals. Serum samples were obtained after 2 weeks of each immunization and IgG levels evaluated by ELISA. For experimental control, animals were immunized with phosphate-buffered saline (PBS) in Alhydrogel (control) and with heat-killed whole-leptospires (bacterin). Unpaired t-test was used to determine statistically significant difference between proteins and PBS. All of them showed significance < 0.05.

**Figure 3:**
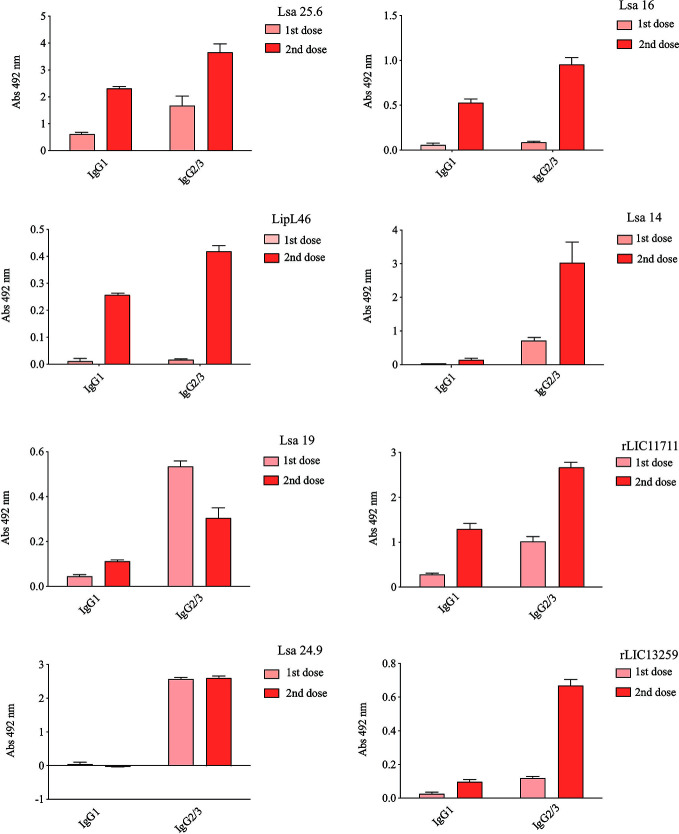
Evaluation of IgG isotypes produced after immunization with each recombinant protein. ELISA plates were coated with each recombinant protein and incubated with increasing concentrations of hamsters’ sera, obtained after immunizations with the respective recombinant proteins. The detection of IgG isotypes was obtained by incubation with HRP-conjugated anti-hamster IgG1 or anti-hamster IgG2/IgG3 (1:5,000) and readings were taken at 492 nm. One-way ANOVA was performed to analyze the difference between IgG1 or IgG2/3 and protein groups. For both cases significance was <0.05.

Hamsters subcutaneously immunized with the Lsa25.6, Lsa16, LipL46, Lsa14, Lsa19, rLIC11711, Lsa24.9, and rLIC13259 proteins adsorbed on alum adjuvant were bled from the retro-orbital plexus 14° day after each immunization; serum samples were obtained, pooled from three animals, and analyzed in triplicate by ELISA to evaluate IgG total production specific for each antigen. The results obtained for each protein are shown in **Figure 2** and refer to two independent immunization assays. Measured IgG levels to each protein were higher for the respective individually vaccinated groups, with higher levels after the second immunization for all the antigens except Lsa19. As observed, bacterin-immunized animals and PBS did not induce IgG levels against each protein. In contrast, bacterin-immunized animals induced IgG level high against killed whole cell antigen, since the first dose given (data not shown). To further evaluate the antibody subclasses elicited by the recombinant proteins, IgG1 and IgG2/3 isotypes were measured. The results show that Lsa25.6, Lsa16, LipL46, Lsa19, rLIC11711, and rLIC13259 induced a mixed response, with both IgG1 and IgG2/3 subclasses being detected, with the latter showing a higher signal. Lsa14 and Lsa24.9 induced exclusively IgG2/3 antibodies (**Figure 3**). The mechanism underlying the absence of IgG1 after immunization with these antigens is not yet clear, but it seems that the presence of IgG subclasses is dependent on several factors, including the structure of the antigen, dose, administration route, and host genetic background (27–30).

### Immunoprotective Profile in Immunized Hamsters After Challenge With Virulent *Leptospira*

**Figure 4:**
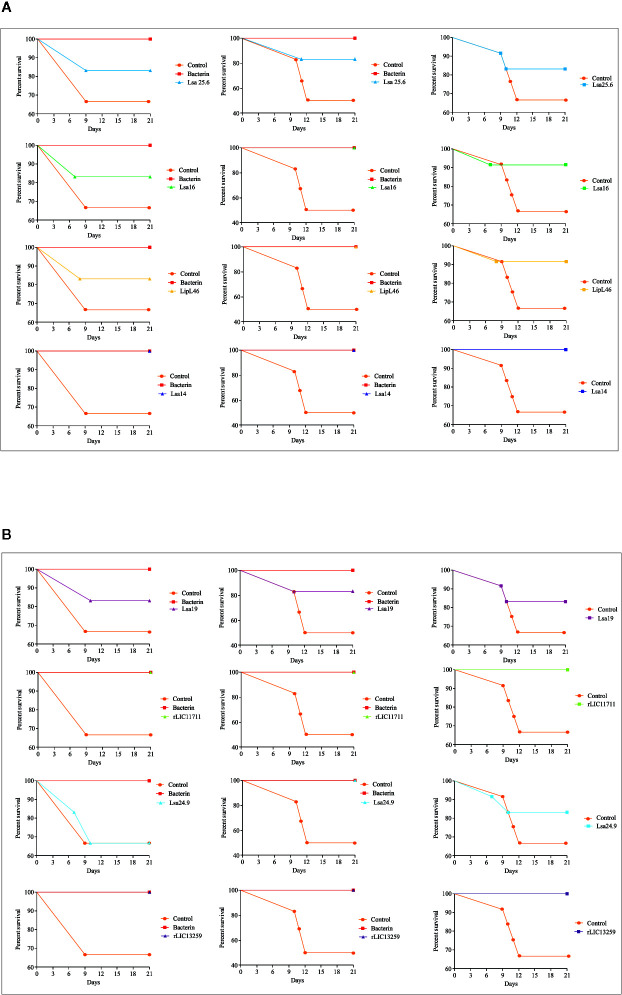
Survival curves of immunized hamsters after challenge with *Leptospira interrogans*. Virulent leptospires (10^4^) were inoculated intraperitoneally in hamsters, previously immunized with recombinant proteins, and monitored daily for 21 days, inspecting the clinical signs of leptospirosis. Animal groups immunized with PBS (control) and bacterin were used as negative and positive controls, respectively. The graphs illustrate the survival curves obtained for Lsa25.6, Lsa16, LipL46, and Lsa14 **(A)** and Lsa19, rLIC11711, Lsa24.9, and rLIC13259 **(B)** in two independent experiments (left and middle) and the average result (right).

**Figure 5:**
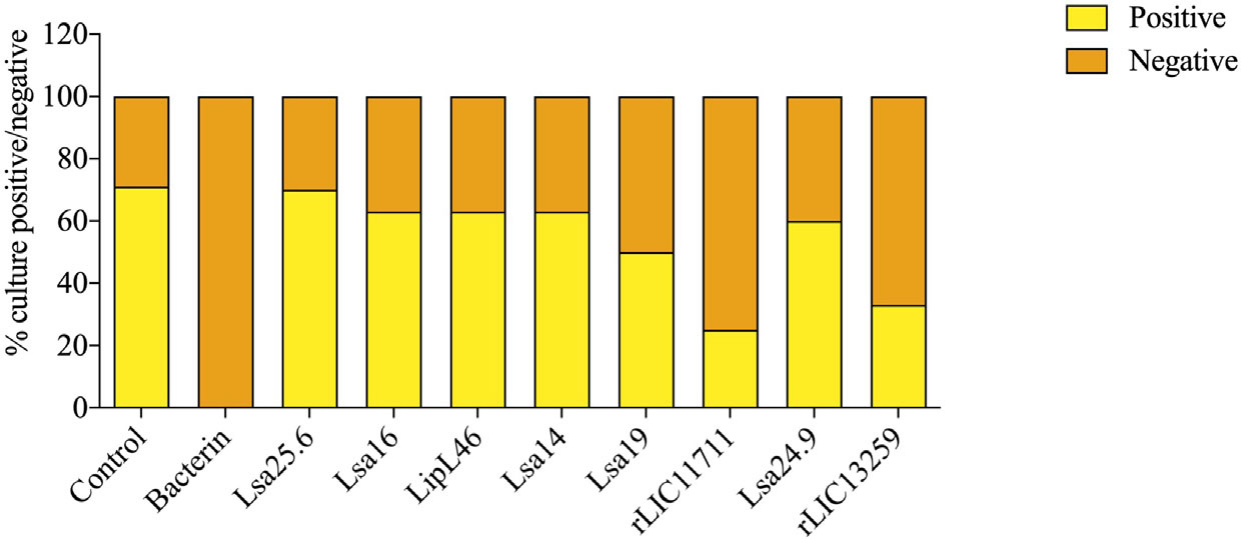
Protective effect elicited in hamster by recombinant proteins assessing the presence or absence of leptospires in animal kidneys after the challenge. Kidneys were removed from euthanized hamsters in 21 days-post challenges with *Leptospira interrogans* and the resulting macerate was incubated in liquid Ellinghausen-McCullough-Johnson-Harris (EMJH) medium and monitored for 30 days. The presence or absence of leptospires in cultures was determined as positive and negative culture, respectively. The graph represents the percentages obtained of two experiments.

To determine whether humoral response elicited by recombinant proteins was able to protect animals from infection by pathogenic leptospires, animal challenge assay was performed in two independent experiments. The data obtained are shown individually for each protein in **Figure 4**. The graphs were plotted with the data from the first and second experiments and the average data. In the first experiment, all proteins, but Lsa24.9, conferred partial protection against challenge when compared to the control. The same percentage of survivors was obtained in the second experiment, except for Lsa24.9. When the average of the two experiments was analyzed, we identified 25 to 42% partial protection since survivors were found in the control group. Variability of results was due to the non-isogenic nature of the hamster model, and it has been observed throughout the literature (10). Currently, we are trying to establish endpoint criteria to avoid this variability. In relation to the ability of leptospires to colonize the renal tubules in these immunized animals, we observed that although there were survivors in the control the majority of them harbored leptospires in their kidneys, in contrast to animals immunized with Lsa19, rLIC13259, and rLIC11711 antigens, which showed the capacity to promote bacterial clearance. **Figure 5** shows a marked difference between survivors of the control group and survivors immunized with these three proteins. While 71% of the control had a positive culture, 50, 33, and 25% of animals respectively immunized with Lsa19, rLIC13259, and rLIC11711 recombinant proteins showed a positive culture (**Table 1**). Although no complete sterilizing immunity was seen in the immunized animals with the recombinant proteins, the understanding of the immune mechanisms involved in that reduction will contribute to the development of effective vaccines. Since all of the antigens tested in this work elicited similarly high levels of IgG1 and IgG2/3 as these three candidate antigens, it is clear that high immunogenicity alone is not sufficient for protection, since the immune response needs to be elicited against specific key antigens. Taken together, these results indicate that recombinant proteins LIC13259 and LIC11711 could induce renal clearance in immunized animals. This potential could be explored by combining these recombinant proteins with other leptospiral antigens or by producing new chimeric antigens.

**Table 1:** Features of survivors immunized with recombinant proteins followed by challenge with *Leptospira interrogans*.

Groups	1° experiment				2° experiment				Average of experiments			
	Survivors/	% Survivors	Positive	% positive	Survivors/	% Survivors	Positive	% positive	Average	%	Culture	%
	Total		Culture	Culture	Total		Culture	Culture				
Control	4/6	66	2/4	50	3/6	50	3/3	100	7/12	58	5/7	71
Bacterin	6/6	100	0/6	0	6/6	100	0/6	0	12/12	100	0/12	0
Lsa25.6	5/6	83	2/5	40	5/6	83	5/5	100	10/12	83	7/10	70
Lsa16	5/6	83	1/5	20	6/6	100	6/6	100	11/12	91	7/11	63
LipL46	5/6	83	3/5	60	6/6	100	4/6	66	11/12	91	7/11	63
Lsa14	6/6	100	4/6	66	5/5^#^	100	3/5	60	11/11	100 *	7/11	63
Lsa19	5/6	83	3/5	60	5/6	83	2/5	40	10/12	83	5/10	50
Lsa24.9	4/6	66	2/4	50	6/6	100	4/6	66	10/12	83	6/10	60
rLIC13259	6/6	100	0/6	0	6/6	100	4/6	66	12/12	100 *	4/12	33
rLIC11711	6/6	100	2/6	33	6/6	100	1/6	16	12/12	100 *	3/12	25

*Fisher’s exact test value is 0.0373.

^#^One animal died at the beginning of the experiment.

## Discussion

The development of effective vaccines against *Leptospira* so far is a challenging endeavor. Several studies have been conducted using recombinant proteins as vaccine candidates, but to date, no ideal candidate has been identified. Data obtained with Lig proteins have demonstrated promising results, with high percentage of survival (31). However, the ability of these vaccines to promote sterilizing immunity has not been achieved. Moreover, LigA protein is not conserved among pathogenic *Leptospira* spp., which reinforces the search for new conserved vaccine antigens. In this study, we evaluated the immunoprotective profile displayed by 8 conserved outer membrane proteins of *Leptospira*, namely in their recombinant form: Lsa25.6, Lsa16, LipL46, Lsa14, Lsa19, Lsa24.9, rLIC13259, and rLIC11711. These recombinant proteins, previously described as potential leptospiral adhesins involved in host-pathogen interactions, were evaluated for their capacity to induce an immune response able to promote survival in hamsters challenged with virulent *Leptospira.* Also, the ability of these proteins promote prevention of leptospires in hamsters` kidneys was inferred by presence or absence of bacteria in cultures.

Vaccines based on virulence factors have been considered to be the best strategy to control pathogenic disease, since the antibodies produced are able to neutralize the target antigen and therefore help in the destruction and removal of the pathogen (32). BibA, a highly conserved antigen, present in bacterial cell and involved in *Streptococcus* virulence is considered a strong vaccine candidate due to its ability to induce opsonizing antibodies (33). Interestingly, not all antibodies require the classical complement pathway to eliminate pathogens. It has been demonstrated that antibodies against OspB of *Borrelia burgdorferi* have bactericidal activity in a complement-independent manner (34, 35), showing the existence of a strategies diversity to remove pathogens from host.

In this study, IgG antibodies were detected in response to all evaluated proteins. However, the IgG levels produced for each protein were not associated with hamster survival rate. Similar results have been observed with other leptospiral antigens when used in a challenge model (10, 15). Intriguingly, Hartwig *et al*. (2013) (36) demonstrated an increased rate of survivors even when no antibodies were detected, suggesting a cellular mechanism. Unfortunately, the lack of immunological reagents to study immune response in a hamster model has hampered our efforts to elucidate the immune mechanism involved in protection against leptospirosis. Therefore, we tried to understand the immune response profile generated by these proteins by evaluating the IgG subclasses, since different subclasses have different effector functions. The regulation of IgG subclass switching by T-helper cells was based on mouse studies, in which Th1 cells were assumed to be associated with the generation of IgG2a (37). Thus, IgG1 and IgG2a isotypes have been used as markers of Th2 and Th1 response in mouse model, respectively. In the leishmaniasis mouse model, a higher IgG2a/IgG1 ratio was associated with protective immune response (38). IgG subclass switching in hamsters is still unclear. Originally, the IgG class in hamsters was divided only into IgG1 and IgG2 subclasses (39). A third subclass, IgG3, has been defined in some hamster strains (40). Accordingly, as observed in mice, Verma and collaborators (41) demonstrated that hamsters challenged with amastigotes of *Leishmania donovani* promoted an upregulation of Th1 cytokine expression and a higher IgG2/IgG3 level, suggesting that the increase in IgG2/IgG3 in hamsters is associated with Th1 immune response. Unfortunately, it was not possible for us to determine a clear association between the survival rate and amount of IgG subclasses produced, since most of the antigens induced both IgG1 and IgG2/3 antibodies. Both IgG subclasses were also found in response to a LigB subunit vaccine, and an increase in protection was observed (13). Curiously, in the same experiment, animals immunized with bacterin, normally used as positive control, induced only IgG2/3 antibodies. A higher level of IgG2/3 antibodies has also been observed in control animals immunized with bacterin (25), in agreement to our results (data not shown). This could suggest the existence of more than one mechanism of action capable of controlling leptospire infection.

In fact, antibodies can have several biological effects against pathogens, such as neutralization, phagocytosis, antibody-dependent cellular cytotoxicity, and complement-mediated lysis (42). In our study, both proteins rLIC11711 and rLIC13259, which showed a greater ability to promote prevention against leptospiral infection, were identified previously as human complement-binding proteins. That ability was specific for both proteins since LigAc, LenA, LcpA, and Lsa23, characterized as factor H-binding proteins, were not able to promote such effect when assayed in an animal model (14). Moreover, previous *in vitro* experiments performed with rLIC11711 and rLIC13259 have suggested that both proteins could promote *Leptospira* resistance to human serum, suggesting their active participation in the host-virulence mechanism (23, 24). Our attempt to determine experimentally if antibodies produced against both proteins developed an opsonizing role, as observed with BibA protein, was unsuccessful due to methodological problems (data not shown). However, we suppose that the antibodies elicited against rLIC11711 and rLIC13259 proteins could be acting in a way to make leptospires vulnerable to the host-immune attack.

The major challenge in the leptospirosis field is the development of vaccines having the capacity to confer broad-spectrum protection against all pathogenic serovars, prevent renal colonization, and induce long-lasting immune protection. Although, our studies have presented survival problems in the control group, Lsa14, rLIC13259, and rLIC11711 showed differences statistically significant after challenge with *L. interrogans* serovar Kennewicki. However, only the rLIC13259 and rLIC11711 proteins showed a greater ability to control leptospires from hamster kidneys. We believe that the interaction of these two proteins with the host complement system may play a role in bacterial clearance. This could shed light on the immune mechanisms involved in leptospirosis protection. Still, more questions than answers remain, and more thorough studies are still needed to elucidate the host immune response that led to bacterial clearance, including cell-mediated immune analysis. In conclusion, this is the first study to show a correlation between proteins involved in host complement system interactions and a greater ability to promote sterilizing immune protection in a hamster model of leptospirosis.

## Data Availability Statement 

The original contributions presented in the study are included in the article/supplementary material. Further inquiries can be directed to the corresponding author.

## Ethics Statement 

The animal study was reviewed and approved by The Ethical Committee for Animal Research of Instituto Butantan approved animals involved in these studies under protocol number 3549261016. The Committee in Animal Research adopts the guidelines of the Brazilian College of Animal Experimentation.

## Author contributions 

All authors contributed to the article and approved the submitted version.

## Funding 

The following Brazilian agencies: FAPESP (grant 2014/50981-0), CNPq (grant 301229/2017-1) and Fundação Butantan, financially supported this work; MC, LK, LF, and AT have fellowships from FAPESP (2018/08131-0; 2018/09652-4; 2017/06731-8 and 2016/11541-0, respectively). The funders had no role in study design, data collection and analysis, decision to publish, or preparation of the manuscript.

## Conflict of Interest

The authors declare that the research was conducted in the absence of any commercial or financial relationships that could be construed as a potential conflict of interest.
